# Minimizing Cardiovascular Adverse Effects of Atypical Antipsychotic Drugs in Patients with Schizophrenia

**DOI:** 10.1155/2014/273060

**Published:** 2014-02-04

**Authors:** Fadi T. Khasawneh, Gollapudi S. Shankar

**Affiliations:** ^1^Department of Pharmaceutical Sciences, College of Pharmacy, Western University of Health Sciences, 309 E. Second Street, Pomona, CA 91766-1854, USA; ^2^Department of Pharmacy Practice and Administration, College of Pharmacy, Western University of Health Sciences, 309 E. Second Street, Pomona, CA 91766-1854, USA

## Abstract

The use of atypical antipsychotic agents has rapidly increased in the United States and worldwide in the last decade. Nonetheless, many health care practitioners do not appreciate the significance of the cardiovascular side effects that may be associated with their use and the means to minimize them. Thus, atypical antipsychotic medications can cause cardiovascular side effects such as arrhythmias and deviations in blood pressure. In rare cases, they may also cause congestive heart failure, myocarditis, and sudden death. Patients with schizophrenia have a higher risk of cardiovascular mortality than healthy individuals, possibly because of excessive smoking, the underlying disorder itself, or a combination of both factors. Increased awareness of these potential complications can allow pharmacists and physicians to better manage and monitor high risk patients. Accurate assessments are very important to avoid medications from being given to patients inappropriately. Additionally, monitoring patients regularly via blood draws and checking blood pressure, heart rate, and electrocardiogram can help catch any clinical problems and prevent further complications. Finally, patient and family-member education, which pharmacists in particular can play key roles in, is central for the management and prevention of side effects, which is known to reflect positively on morbidity and mortality in these patients.

## 1. Introduction and Discussion

The risk of cardiovascular-related morbidity and mortality is known to increase in patients with schizophrenia. Patients with schizophrenia have an increased risk of sudden death and are 2–4 times more likely to die prematurely compared to the general population [1]. The second generation antipsychotics (SGAs) are associated with cardiovascular side effects that can have serious consequences to patients [1–3] (Table 1). Atypical antipsychotic drugs are a chemically diverse group of drugs, and the type and the degree of associated cardiovascular effects may therefore vary. Risk factors for cardiovascular adverse effects associated with the use of atypical antipsychotic drugs include advanced age, autonomic dysfunction, preexisting cardiovascular disease, female gender (for risk of QTc interval prolongation and torsade de pointes), electrolyte imbalances (particularly hypokalemia and hypomagnesemia), elevated serum antipsychotic drug concentrations, genetic characteristics, and the psychiatric illness itself.

Antipsychotics have been used for more than five decades for the treatment of psychosis, which can result from a number of disorders including bipolar disorders, delirium, delusional disorders, psychotic depression, paranoia, schizophrenia, Tourette's syndrome, and substance-induced psychosis and various neurologic conditions such as dementias, Huntington's disease, multiple sclerosis, and parkinsonism. The first generation antipsychotic medications (FGAs), also commonly referred to as “neuroleptics,” which include phenothiazines, butyrophenones, and thiothixenes, treat many of the symptoms of psychosis but have undesirable side effects such as akathisia, tardive dyskinesia, dystonia, and parkinsonism, collectively referred to as “extrapyramidal side effects” (EPS). The newer atypical antipsychotics (SGAs; Table 1) have been shown to treat the positive symptoms such as psychosis and the negative symptoms such as apathy and withdrawal and also have been shown to have a beneficial effect on cognition with a reduced risk of EPS.

As indicated before, it is well established that patients with mental disorders have a higher prevalence of modifiable risk factors for cardiovascular disease that may include obesity, hypertension, diabetes mellitus, and dyslipidemia. For example, obesity can be 1.5 to 2 times more prevalent in people with schizophrenia and affective disorders than in the general population [4]. Recent findings suggest that psychotropic medications used to treat this population also contribute to weight gain [5]. In a five-year naturalistic study of patients treated with clozapine, a researcher found that weight gain increased until 46 weeks after drug initiation [6]. Added to other cardiovascular risks such as sedentary lifestyle, obesity, substance abuse, and smoking that psychiatric patients are more prone to, there is clearly a higher rate of cardiovascular mortality associated with this population. Current treatment guidelines by the American Psychiatric Association recommend that patients who have preexisting heart conditions or other cardiovascular risks be assessed for their cardiovascular risk before antipsychotic therapy is initiated and routinely monitored thereafter [7].

The association between SGAs and hypertriglyceridemia has been established in wide range of studies [8]. In addition to age, sex, and low levels of high-density lipoproteins (HDL), high plasma TG is an independent risk factor for the development of coronary atherosclerosis and coronary heart disease (CHD). Due to the increased prevalence of heart disease and associated mortality in the schizophrenia population [9], the potential for SGAs to increase TG is an important concern. The mechanism of hyperlipidemia due to SGAs is still unclear but the condition is more prevalent among those who are overweight or obese. Although all atypical antipsychotics (with the exception of ziprasidone and aripiprazole) increase serum TG to some degree, severe hypertriglyceridemia occurs predominantly with clozapine and olanzapine. Moreover, in one small study, two patients prescribed quetiapine experienced a 48% increase in TG levels. It is noteworthy that both hyperlipidemia and hypertriglyceridemia are thought to be associated with insulin resistance. In extreme cases of hypertriglyceridemia (i.e., development of pancreatitis), it may be associated with decreased production of islet cells and decreased glucose utilization.

In summary, adverse reactions to drugs (along with suboptimal treatment) are very costly and cause pain and suffering for the patient, resulting in potential disability or sometimes death [10]. In fact, in one study, 17% of the problems were a result of inappropriate drug therapy [11]. Consequently, knowledgeable, skilled pharmacists are essential for the safe, timely, effective, and efficient care of patients. In this connection, in mental health facilities, pharmacy specialists can contribute significantly to such tangible results as

1. optimal use of drugs,
2. substantial reduction of adverse drug reactions,
3. fewer complications related to drug treatment,
4. reduced morbidity and mortality by minimizing medication-related problems,
5. improved laboratory monitoring of drug therapy,
6. improved patient satisfaction.

Integrating the activities of traditional pharmacy practice (e.g., dispensing), pharmacist clinician activities require an understanding of the advancements made in collaborative practice and cognitive services. Cognitive service is a component of pharmaceutical care that pharmacists have been providing for many years. Cognitive service is defined as services provided by the pharmacist for the patient or other health care professionals for the purposes of promoting optimal health and/or drug therapy, not necessarily drug product or drug distribution related [12]. These services primarily focus on optimizing a patient's drug therapy and ensuring appropriateness, safety, and efficacy. Cardiovascular monitoring in a hospital or in a clinic by a pharmacist is a specialized service.

Mechanisms for assuring the competence of specialist pharmacists to monitor cardiovascular side effects in our opinion may include but not limited to the following:

1. to be a BCPP (board certified in psychiatric pharmacy) or its equivalent training or experience like residency in psychopharmacy and/ or BCPS with psychiatry background;
2. to be competent to interpret ECG (Lead II);
3. to be trained to take vitals and listen to and interpret heart sounds S1 and S2;
4. to be able to interpret laboratory reports accurately.

A professional function of a pharmacist is to provide drug therapy directed at definite outcomes that improve quality of life for patients. The profession of pharmacy has an obligation to the public to ensure that pharmacists provide safe, effective, timely, and compassionate care to patients. However, specialized areas may require specialized knowledge and skills, attained only through additional training.

Based on these considerations and given the shortage of primary care practitioners in general and in mental health/psychiatric facilities in particular, the present paper not only underscores but also provides guidance for the management of cardiovascular complications/manifestations in patients receiving treatment for schizophrenia.

## 2. Blood Pressure Changes

### 2.1. Hypotension and Orthostatic Hypotension

The definition of orthostatic hypotension is the decrease of 20 mmHg or more in systolic pressure or the decrease of 10 mmHg or more in diastolic pressure within three minutes of standing [13]. Orthostatic hypotension is a common side effect of atypical antipsychotics. It is caused by anticholinergic or alpha-1 adrenoceptor blockage [14]. Alpha-1adrenoceptors cause vasoconstriction in certain vascular beds. The blockade of these receptors leads to vasodilation which causes blood pressure to decline. This effect is mostly seen with blood pressure in the standing position, when sympathetic tone is important to maintain adequate blood pressure (Table 2). Prolonged effect of orthostatic hypotension has been associated with adverse outcomes such as stroke or myocardial infarction in severe cases.

Hypotension, including orthostatic hypotension, is a significant side effect encountered with atypical antipsychotic drugs. Adrenergic blockade may result in number of risks, such as syncope, falls, fractures, increased anginal episodes, and orthostatic hypotension. The agents that most commonly cause hypotension include clozapine, quetiapine, and risperidone. Olanzapine does not block alpha adrenergic receptors and has not been linked to orthostatic hypotension, although dizziness has been reported in some patients. Risperidone can occasionally cause orthostatic dizziness, hypotension including orthostatic hypotension, and reflex tachycardia. The least hypotensive effects are reported with ziprasidone. Atypical antipsychotic drugs in combination with cardiovascular medications such as methyldopa, diuretics, adrenergic blockers, calcium antagonists, angiotensin-converting enzyme inhibitors, angiotensin-II receptor blockers, nitrates, and others may aggravate the hypotensive effects.

Orthostatic hypotension can contribute to the increase in risk for injury (i.e., hip fractures) and falls in the elderly population. This is because the elderly are frailer and have a higher incidence of osteoporosis compared to the younger population. It is recommended that those who take these psychotropic medications should be aware of dizziness. Recommendations of taking appropriate orthostatic blood pressure assessment are to have the patient lie supine for ten minutes, obtain blood pressure and heart rate, then take blood pressure and heart rate immediately after the patient arises, and ask about dizziness [15]. Heart rate assessment will aid in differential diagnosis. Heart rate increases by 10–15 bpm normally on rising; in orthostatic hypotension, heart rate may increase (with a concomitant fall in blood pressure) within a range of 15–30 bpm. Next, have the patient maintain an upright posture for three minutes; then obtain blood pressure and heart rate again. Table 2 summarizes the key points for the assessment, monitoring, and patient education regarding orthostatic hypotension in antipsychotic-treated patients.

### 2.2. Hypertension

The SGAs that are associated with hypertension include clozapine, olanzapine, and ziprasidone. Quetiapine and risperidone appear to have the lowest risk of hypertension. Assessing the patient's pre-existing illness and/or monitoring the patient's blood pressure and heart rate at baseline and then periodically during treatment can help control and prevent further complications from arising.Table 2 summarizes the key points for the assessment, monitoring, and patient education regarding hypertension in antipsychotic-treated patients.

## 3. QTc Interval Prolongation and Torsades de Pointes

An electrocardiogram (ECG) measures the electrical activity of the heart and can serve as a diagnostic aid to determine possible heart complications. The phases of the ECG are summarized in Table 3. “P” waves show electrical currents moving towards the atrium of the heart, causing depolarization. When this occurs, there is a rapid influx of sodium ions that move into the cells through the sodium channel, while potassium ions are slowly moving out of the cell in the atria. It then produces a QRS complex that shows currents going to the ventricles causing depolarization. Similar to the “P” wave, sodium ions move into the cells while potassium ions are moving slowly out of the cell but that is in the ventricles. The calcium influx that follows causes the plateau of the action potential and helps myocardial contraction. The depolarization slows down in the ST segment. Finally, the “T” wave and “U” wave are associated with ventricular repolarization. The normal QTc intervals are approximately 400 msec or less. The greater the duration is, the more likely the ventricular arrhythmias may occur, especially if the interval is greater than 500 msec.

**Figure 1:**
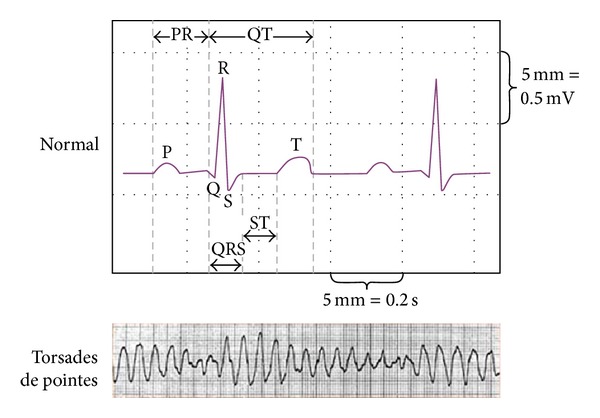
Normal versus torsades de pointes ECG.

Torsades de pointes (TdP) is a polymorphic ventricular tachycardia associated with a prolonged QTc interval. The ECG pattern is distinctive and is called *twisting* because the peaks are at their smallest in one lead, and largest in another lead (Figure 1). Torsades de Pointes is often self-limiting, but when sustained can cause ventricular fibrillation and sudden death. Risk factors for TdP are female sex, history of heart disease, presence of a QT interval prolonging agent, hypokalemia, history of QT prolongation, family history of QT prolongation, QTc > 450 ms at baseline, and bradycardia [16] (Table 4). Patients who have these risk factors should be monitored carefully or be taken off the drug entirely.

Potassium channels play an important role in ventricular arrhythmias (i.e., torsades de pointes). The potassium channel that is most involved in drug-induced QT syndromes is the potassium rectifier (Ik_r_) channel. Although olanzapine, quetiapine, and risperidone bind to the Ik_r_ channel, it is unclear whether they cause TdP or not. Some of the psychotropic drugs that can possibly cause TdP are listed in Table 5 and include chlorpromazine, haloperidol, pimozide, thioridazine, mesoridazine, and ziprasidone.

The first generation antipsychotic thioridazine, even at therapeutic dosages, is frequently cited as causing TdP and sudden death by blocking the potassium channel Ik_r_ and prolonging the QT interval [17]. It is recommended that the lowest dose possible be given to patients with cardiovascular problems because higher doses could cause repolarization abnormalities. This medication is contraindicated in patients who are at risk for TdP.

Ziprasidone has some effect on repolarization, but it is not dose dependent. It prolongs QT interval more than haloperidol, olanzapine, quetiapine, and risperidone but less than sertindole and thioridazine [18]. In premarketing trials, an increased risk of arrhythmia was not associated with ziprasidone; however, this does not assure complete safety from causing any arrhythmias [19].

In one study, the cardiac effects of haloperidol, olanzapine, quetiapine, risperidone, thioridazine, and ziprasidone were compared. Results showed that thioridazine had the longest QTc interval prolongation, followed by ziprasidone, quetiapine, risperidone, olanzapine, and haloperidol (35.6 ms, 20.3 ms, 14.5 ms, 11.6 ms, 6.8 ms, and 4.7 ms, resp.) [18]. Not all drugs with longer QTc interval have a higher risk of torsades. For example, risperidone has a higher QTc interval compared to haloperidol, but haloperidol has been known to cause TdP and sudden death more than risperidone [17]. Measuring the QTc interval of patients is a way to determine the risk of having torsades; however, it should not be used as the only determining factor.  Table 6 lists the relative risk of QTc interval prolongation with common antipsychotic agents.

In this connection, there have been case reports providing evidence for the association of ziprasidone with torsades de pointes [20, 21]. For example, in one case, a 30-year-old woman with a medical history of mental retardation and depression with psychotic features was found unresponsive by emergency medical technicians. Since her regular medications included ziprasidone, after examination, it was concluded that the ziprasidone may have contributed, in part, to her torsades de pointes episode [20]. In a separate case study, a 28-year-old Hispanic woman with a history of mental diseases and on multiple medications including ziprasidone and lithium was admitted to the intensive care unit (ICU). Upon evaluation, her QT interval was found to be prolonged. Given that the patient was on lithium, ziprasidone, and hypokalemic on presentation, which are all risk factors for QT-interval prolongation and progression to torsades de pointes, the episode of torsades de pointes was attributed only in part to ziprasidone [21].

Assessment and monitoring of patients are needed to determine their wellness. Determining the patient's history of heart disease (including family members) and inquiring about other types of psychotropic, heart, or over-the-counter medications are needed to start any drug therapy. A thorough physical examination and ECG monitoring are important in determining the heart rate and blood pressure. Lastly, taking blood samples periodically is needed to determine patient's potassium levels. It is important because low levels of potassium can increase the risk of TdP. Table 2 summarizes the key points for the assessment, monitoring, and patient education regarding arrhythmias/torsades de pointes in antipsychotic-treated patients.

## 4. Sudden Death

There is no known mechanism on how sudden death occurs, but it is known that it may possibly be caused by cardiac arrhythmias, notably TdP following ventricular fibrillation. The common feature is delayed repolarization of the myocardium which causes a prolongation of the QT interval of the ECG. This leaves the myocardium vulnerable to ventricular tachycardia and in some serious cases sudden death.

A study by Ray and his colleagues followed nearly 0.5 million Tennessee Medicaid enrollees for 2.5 years [22] and found 1,487 sudden deaths due to heart problems, or an incidence of 11.6/10,000 person-years. Patients who received antipsychotic drugs, in doses of more than 100 mg of thioridazine or its equivalent, had 2.39 times higher risk of having sudden death compared to nonusers (95% CI = 1.77–3.22). It has been shown that risperidone can prolong QTc interval although reports of sudden death are rare [23]. Sudden death can be contributed to various physical and psychological factors: age, pre-existing heart condition, frequency of prolonged smoking, generic disorder of calcium channels, metabolism of antipsychotic drug, schizophrenia itself, and the use of drugs such as thioridazine, droperidol, haloperidol, pimozide, and sulpiride. There is also some evidence that shows schizophrenic patients having higher risk for sudden death regardless of taking any medications. This increased risk of sudden death can be due to the schizophrenic illness itself or a combination of other factors such as lifestyle (e.g., smoking, poor diet, and general neglect of health) and poor access to medical care [24].

## 5. Myocarditis

Myocarditis, inflammation of the heart muscle, is a rare complication of clozapine therapy. In one study, the estimated incidence was between 0.7% and 1.2% of clozapine-treated patients [25]. The mechanism is unknown but it is suggested that it could be IgE-mediated hypersensitivity to exposure to chemicals or certain medications. When the heart muscle becomes inflamed and weakened, it causes lymphocytic infiltration and other symptoms similar to heart failure, which then may mimic a heart attack. However, it rarely causes sudden death. Symptoms of myocarditis are similar to congestive heart failure. They include fever, chest pain, joint pain or swelling, abnormal heart beats, fatigue, shortness of breath, fainting, low urine output, leg swelling, and the inability to lie flat. Sometimes, no symptoms are present at all.

Myocarditis can be detected from a physical exam showing irregular and/or abnormal heart beats or sounds, fluids in the lungs, skin, and/or the legs. Other examinations include ECG, chest X-ray, WBC, RBC, and blood cultures for infections. Treatment includes an evaluation and treatment of the underlying problem. This may require use of antibiotics for infection and low-salt diet to avoid retaining water. Diuretics are also given to remove body water. Steroids and anti-inflammatory medications are used to reduce inflammation. Abnormal heart rhythm may require the use of additional medications, a pacemaker, or even a defibrillator. If a blood clot is present in the heart chamber, anticoagulants may be given as well. It is important to treat as soon as possible to prevent complications such as heart failure, pericarditis, or cardiomyopathy.

Clozapine, which is (the only drug) indicated for resistant schizophrenia, has a “black box” warning for causing an increased risk of fatal myocarditis. One study reported 23 cases (20 men, three women; mean age 36 years) of clozapine-associated cardiovascular complications: 15 of myocarditis and eight of cardiomyopathy [26]. Of the 23 patients, six died (five deaths from myocarditis). All cases of myocarditis occurred within three weeks of starting clozapine. Of the eight cases of cardiomyopathy, the condition was diagnosed up to 36 months after initiation of clozapine. Necropsy findings showed mainly eosinophilic infiltrates with myocytolysis (consistent with an acute drug reaction). Therefore, clozapine therapy may be associated with potentially fatal myocarditis and cardiomyopathy in young adults with schizophrenia. Clozapine should be discontinued if a patient has an onset of fatigue, dyspnea, tachypnea, fever, chest pain, palpitations, heart failure symptoms, arrhythmias, or ECG abnormalities. Patients should be aware of these side effects so they can let their physicians know right away to discontinue the medications. Table 2 summarizes the key points for the assessment, monitoring, and patient education regarding myocarditis in antipsychotic-treated patients.

## 6. Conclusion

Antipsychotic medications can cause various types of cardiovascular complications (e.g., arrhythmias, hypertension, myocarditis, and orthostatic hypotension). Increased awareness of these potential complications can allow pharmacists and clinicians to better manage and monitor at-risk patients. Accurate assessments are very important to avoid medications from being given to patients inappropriately. In addition, monitoring patients regularly via blood draws and checking blood pressure, heart rate, respiration rate, and ECG can also help catch any clinical problems as well as prevent further complications from occurring. Lastly, educating patients, family, and caregivers on what to look out for and how to handle certain side effects is very important. By doing so, the risk of mortality for patients with mental and behavioral disorders may be further reduced. Pharmacists practicing in mental health facilities, by virtue of their job functions and responsibilities, are in a unique position to play a pivotal role in minimizing the cardiovascular adverse effects of antipsychotic drugs.

## Figures and Tables

**Table 1:** Cardiovascular side effects of second generation antipsychotics.

Drug	Orthostatic hypotension	Hypertension	Myocarditis	Torsades de pointes	Reflex tachycardia
Aripiprazole (Abilify)	X	X			X
Asenapine (Saphris)	X				X
Clozapine (Clozaril)	X	X	X*	X*	X
Iloperidone (Fanapt)	X				X
Lurasidone (Latuda)	X	X			X
Olanzapine (Zyprexa)	X				X
Paliperidone (Invega)	X				X
Quetiapine (Seroquel)	X			X*	X
Risperidone (Risperdal)	X			X*	X
Ziprasidone (Geodon)	X	X		X*	X

*indicates that this side effect is “rare.”

**Table 2:** Assessment, monitoring, and patient education regarding orthostatic hypotension, hypertension, arrhythmias/torsades de pointes, and myocarditis in antipsychotic-treated patients.

Side effect process/step	Orthostatic hypotension	Hypertension	Arrhythmias/torsades de pointes	Myocarditis
Assessment	(1) Elderly (>65 years of age) (2) Risk of osteoporosis or have osteoporosis (3) Disorders that predispose to orthostasis or fall risk (e.g., dementia, gait disorder, and parkinsonism) (4) Assess baseline blood pressure and heart rate in supine and standing positions prior to starting drug therapy (5) History of injury (i.e., hip fractures) or falls (6) Taking any types of over-the-counter medications that may cause dizziness (e.g., allergy remedies, antihistamines, cold remedies, or sleep aids)	(1) Assess baseline blood pressure, heart rate, and respiratory rate prior to starting drug therapy (2) History of hypertension, heart disease, diabetes, and dyslipidemia (3) History of smoking (4) Family history of hypertension and heart disease (5) Taking any type of illicit or over-the-counter medications with vasoconstrictive properties (e.g., decongestant, weight loss supplement, diet pills, or street drugs)	(1) Female gender (2) History of heart disease (3) Using a QT prolonging agent (4) Have hypokalemia (K^+^ < 3.5 mEq/L) (5) Using high dose of offending drug (6) History of QT prolongation (7) Family history of QT prolongation (8) QTc > 450 msec as a baseline prior to drug therapy (9) Have bradycardia (10) Check baseline ECG prior to drug therapy (11) Check blood pressure and heart rate baseline prior to drug therapy (12) Check electrolytes baseline prior to drug therapy	(1) History of recent viral, bacterial, or parasitic infections (2) Allergy to clozapine or any type of medications (3) History of heart failure, cardiomyopathy, pericarditis, or heart attack

Monitoring	(1) Check blood pressure periodically (2) Check heart rate periodically	(1) Check blood pressure periodically (2) Check heart rate periodically	(1) Check ECG periodically for abnormalities (i.e., QT prolongation) (2) Check electrolytes periodically (i.e., sodium, potassium, and calcium) (3) Check blood pressure and heart rate periodically	(1) Check ECG and chest X-ray periodically as needed (2) Do blood draws periodically (i.e., WBC, RBC) (3) Monitor levels of clozapine periodically (4) Test for blood cultures for detecting any infection (5) Check for heart rate, heart beat, and sounds (6) Check for edema in the arms, legs, and lungs

Patient education	(1) Take calcium and vitamin D supplement for bone health and strength (2) Arise slowly from bed or chair when getting up (3) Ask for assistance when having difficulty standing up (4) Medication can cause dizziness and palpitations (5) Avoid or limit the amount of alcohol beverage intake (6) Consult your doctor or pharmacist prior to purchasing any over-the-counter medications	(1) Avoid excessive sodium salt intake (2) Exercise regularly (at least 30 minutes/day for 5 days) (3) Try to quit smoking or smoke less (4) Avoid or limit the amount of alcohol beverage intake (5) Consult your doctor or pharmacist prior to purchasing over-the-counter medications	(1) Let your doctor know if your heart rate is very fast or very slow (2) Check your heart rate and blood pressure regularly (3) Ask your doctor prior to purchasing over-the-counter medications (4) Maintain a well-balanced diet (5) Try to quit smoking or smoke less	(1) Let your doctor know if you have fever, chest pain, joint pain or swelling, abnormal heart beats, fatigue, shortness of breath, fainting, low urine output, leg swelling, and the inability to lie flat (2) Check your heart rate regularly

**Table 3:** Phases of the electrocardiogram (ECG).

Wave	Electrical activity
P wave	Atrial depolarization
PR interval	Time between the onset of depolarization in the atria and the onset of depolarization in the ventricles
QRS complex	Ventricular depolarization
ST segment	Plateau phase of ventricular depolarization
T wave	Ventricular repolarization
QT interval	Ventricular depolarization and repolarization
U wave	A normal component of the surface ECG represents the delayed repolarization of the Purkinje network, seen at the same time as early after depolarization in patients with a prolonged QT interval and TdP

TdP: torsades de pointes, a malignant form of ventricular arrhythmia, is polymorphic.

**Table 4:** Risk factors contributing to QTc interval prolongation and torsades de pointes.

Risk factor	Causes/implications
Sex (female)	QT intervals longer in women than in men QT interval longer during first half of menstrual cycle

Age (elderly)	Comorbid coronary artery diseases Multiple medications Pharmacokinetic/pharmacodynamic changes

Electrolyte imbalance Hypokalemia, hypomagnesemia Hypocalcemia	Diuretic use Excessive vomiting or diarrhea Postprandial hypokalemia

Congenital long QT syndrome	Associated with torsade and sudden death

Cardiac disease with history of acute or chronic myocardial ischemia, CHF, cardiac arrhythmias, and bradycardia	Increased risk of cardiac arrhythmias

Drugs known to prolong QTc interval	May potentiate QTc prolongation

Medication overdose with drugs that prolong the QTc interval	QTc prolongation generally dose dependent

Concomitant medications, liver disease	Adverse events with cytochrome P-450 enzyme system inhibition, leading to increased drug levels that can increase QT interval

Endocrine/metabolic disorders Diabetes, obesity Hypothyroidism, pituitary insufficiency	Via electrolytes or cardiovascular disease

Injury to the central nervous system Stroke, infection, and trauma	Via autonomic nervous system dysfunction

**Table 5:** Antipsychotropic medications with potential risk for QTc interval prolongation.

Typical antipsychotics	Atypical antipsychotics
Pimozide (Orap)	Quetiapine (Seroquel)
Chlorpromazine (Thorazine)	Risperidone (Risperdal)
Haloperidol (Haldol)	Ziprasidone (Geodon)
Thioridazine (Mellaril)	
Mesoridazine (Serentil)	

Adapted from http://www.torsade.org/.

**Table 6:** Relative risk of QTc interval prolongation with common antipsychotic agents.

Risk level	Agent
ECG required or strongly recommended before prescribing (most commonly associated with QTc interval prolongation and torsade de pointes)	Thioridazine Mesoridazine Droperidol Pimozide Haloperidol in large doses IV (commonly ≥100 mg/d)

Mild to moderate risk of QTc interval prolongation (~20 ms) when prescribed alone or with a metabolic inhibitor	Quetiapine Ziprasidone Chlorpromazine

Little or no risk of QTc interval prolongation (~20 ms) when prescribed alone or with a metabolic inhibitor	Haloperidol (oral) Olanzapine Risperidone Clozapine
